# Supplementary material for: Systems approaches identify the consequences of monosomy in somatic human cells
Source: Nat Commun. 2021 Sep 22;12:5576. doi: 10.1038/s41467-021-25288-x (PMC8458293; doi:10.1038/s41467-021-25288-x)
Supplement: Supplementary file 1 — Supplementary Information [file 41467_2021_25288_MOESM1_ESM.pdf]

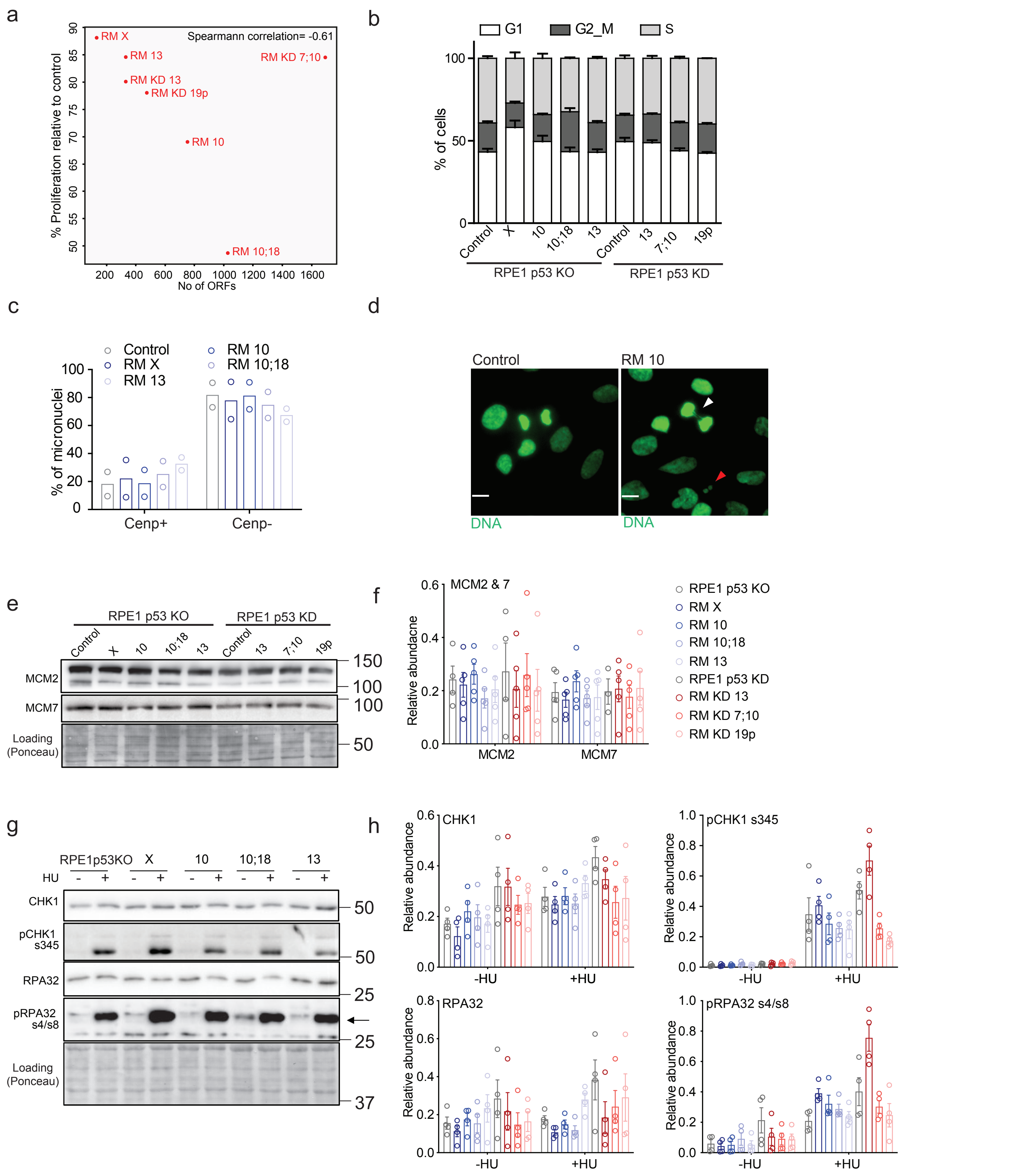

Supplementary figure 1 **Characterization of monosomic cell lines**

a. Correlation between the proliferation and number of open reading frames (ORFs) on the monosomes. The number of ORFs was obtained from NCBI database; the estimated number of non-compensated ORFs was considered for chromosome X. **b**. Cell cycle profiles of control and monosomic cell lines.Bars display mean+SEM percentage of cells in different cell cycle phases of three independent experiments. The representative gating strategy in shown in Sup. Fig 9
c. Fraction of micronuclei positive/negative for CENP signal of two independent experiments. Number of micronuclei analyzed (Control= 84,RM X= 82, RM 10= 86, RM 10;18= 132, RM 13= 77) **d**. Representative image of anaphase bridges in diploid and monosomic cell lines. Scale bar – 10 µm.**e**. Representative immunoblot of the subunits of the key replicative helicase MCM2-7. **f.** Quantification of protein abundance from e. Bars represent the mean+SEM of five independent experiments. **g**. Representative immunoblot of key DNA damage response factors and
h. Quantification of their abundance. Bars represent the mean+SEM of five independent experiments. Arrow points to the specific band for pRPA32 s4/s8. HU-hydroxyurea. Source data are provided as a Source Data file.

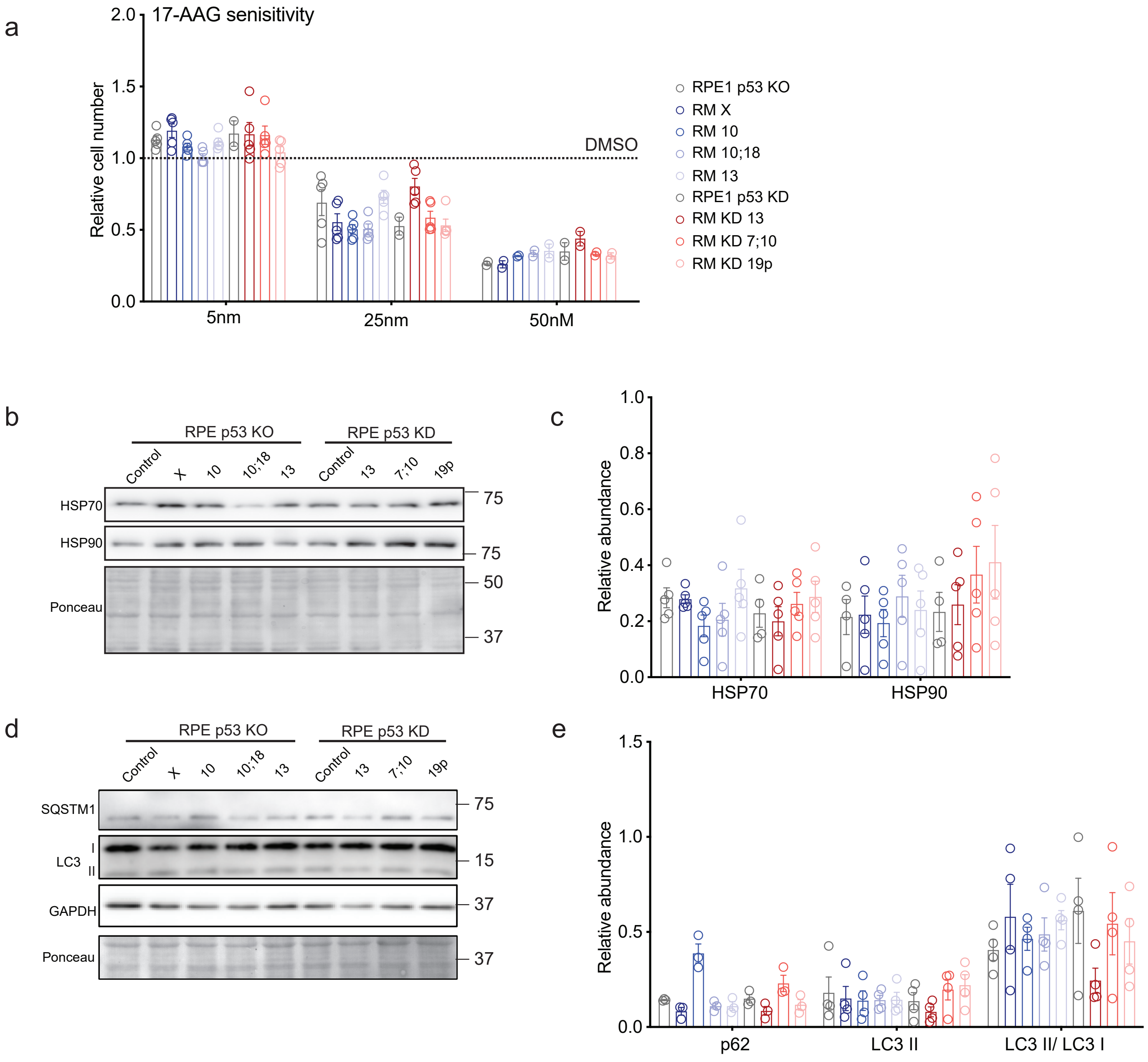

Supplementary figure 2 **No apparent proteotoxic stress in monosomies.**

a. Sensitivity to 17AAG measured by Cell Titer Glo assay. All the values were normalized to DMSO control. Bars display mean±SEM of five independent experiments for 5nm and 25nm and two experiments for 50nm.
b. Immunoblotting of heat shock proteins and **c.** quantifications of at five independent experiments. Bars display. mean±SEM. **d.** Immunoblotting of autophagy related proteins and **e.** quantification of four independent experiments. Bars display Mean±SEM. Ponceau staining was used as a loading control in (**b**) and (**d**). Source data are provided as a Source Data file.

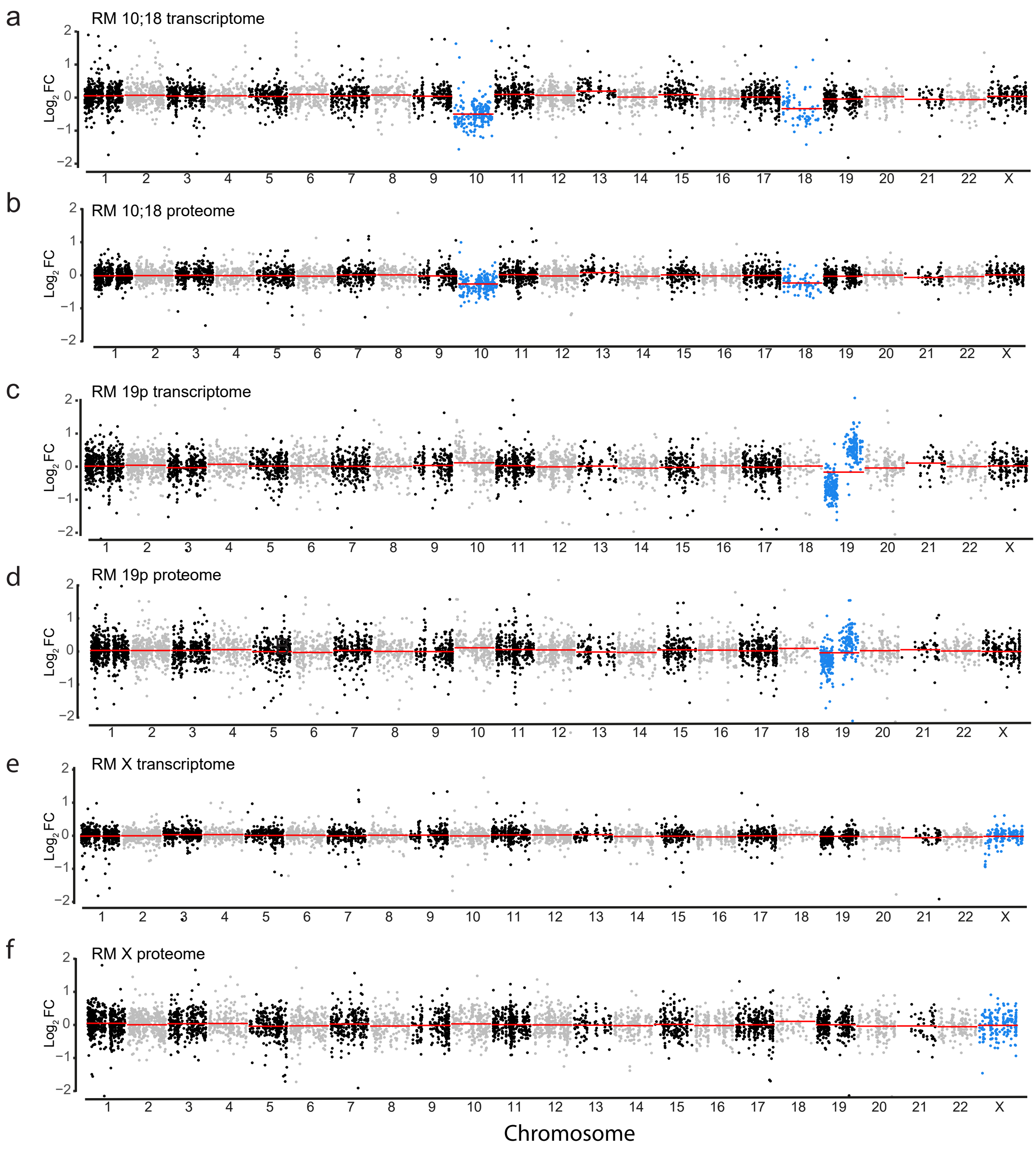

Supplementary figure 3 **Transcriptome and proteome of monosomic cell lines.**

a-f. The relative abundance of mRNAs and proteins of RM10;18, RM 19p and RM X normalized to diploid control and plotted according to the chromosome location of the corresponding genes. The monosomic chromosomes are marked in blue. Red line depicts the median for each chromosome.

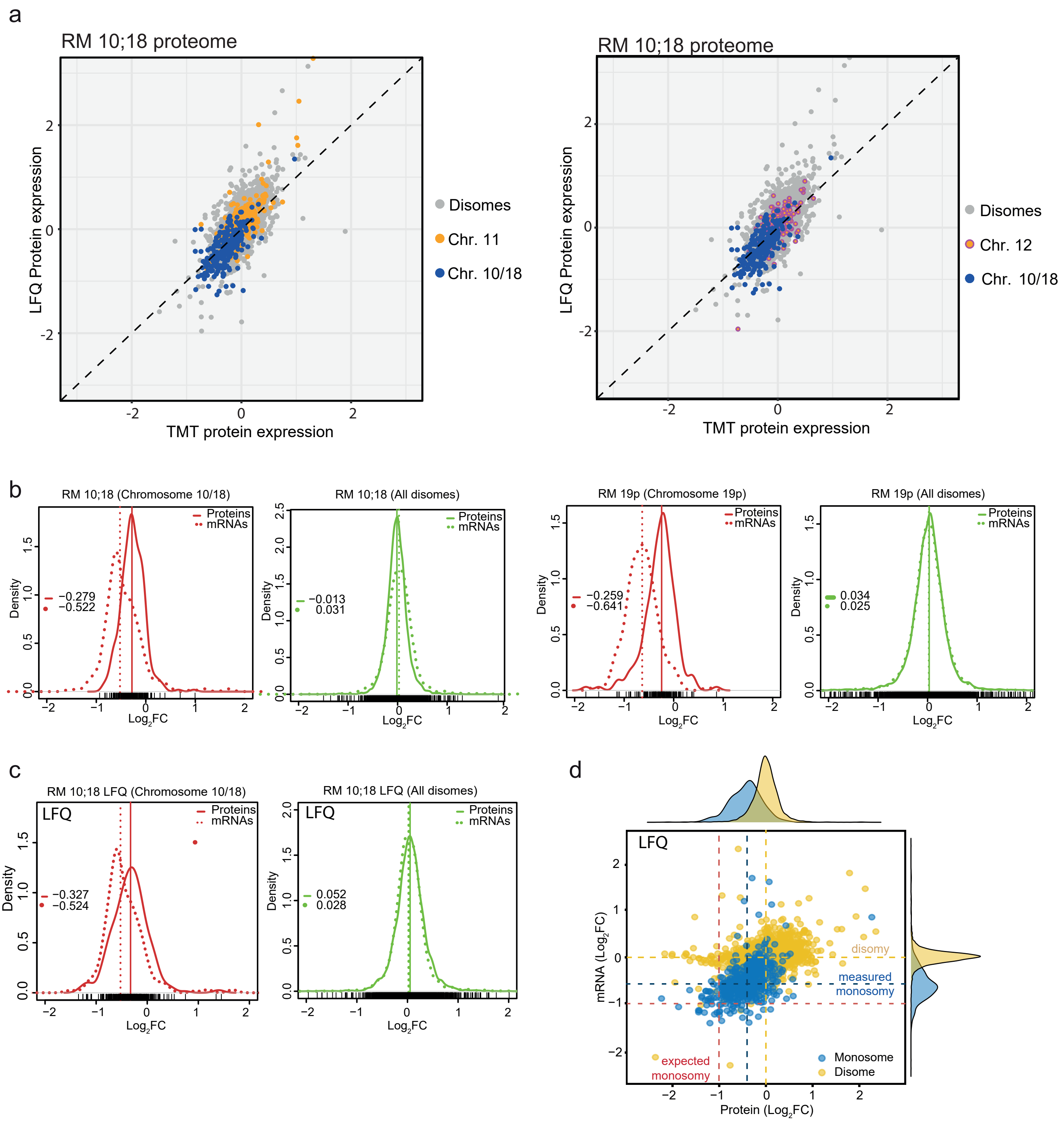

Supplementary figure 4. **Comparative proteome values obtained by LFQ and TMT proteomics analysis.**

a. Scatter plots of the relative protein abundance values obtained by LFQ and TMT proteomics show a strong similarity between the results obtained by these two approaches. RM10;18 proteome is shown, proteins encoded on chromosomes 10 and 18 (monosomic) and 11 and 12 (disomic) are highlighted. All other disomic chromosomes are shown in light gray. **b.** Overlay of mRNA and protein density histograms for RM 10;18 and RM 19p. Red line depicts the monosomes, green line the disomes. **c.** Overlay of mRNA and protein density histograms for RM 10;18 using the LFQ data. **d.** Scatter plot showing the log2 fold change (FC) of mRNA and proteins encoded on monosomes (blue) and disomes (yellow), as in Fig. 3d, but calculated based on the LFQ data. The marginal density histograms show the distribution of respective mRNAs and proteins. The expected median fold change of monosomic genes is marked by red dashed lines. The measured median fold changes of monosomic and disomic genes is marked by blue and yellow dashed lines, respectively.

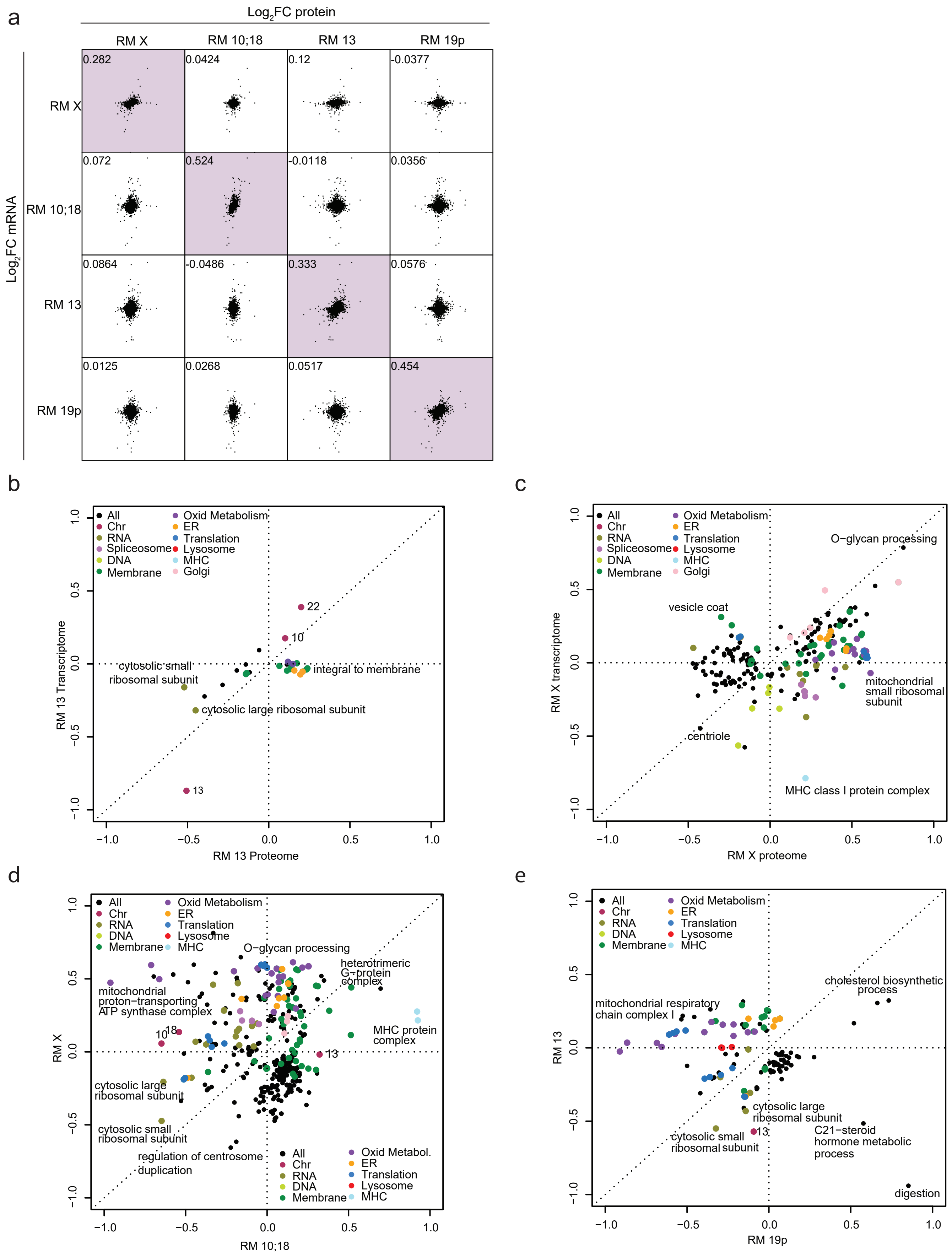

Supplementary figure 5 **Pathway enrichment analysis of monosomies**

a. Scatter plots depicting the Spearmann rank correlation coefficient between the relative abudances of mRNA and proteins for all monosomies. The numbers in each plot represent the Spearmann correlation coefficient. **b,c.** 2D pathway enrichment analysis of transcriptome and proteome of RM 10;18 and RM X. **d,e.** 2D pathway enrichment analysis comparing the proteome of individual monosomic cell lines.

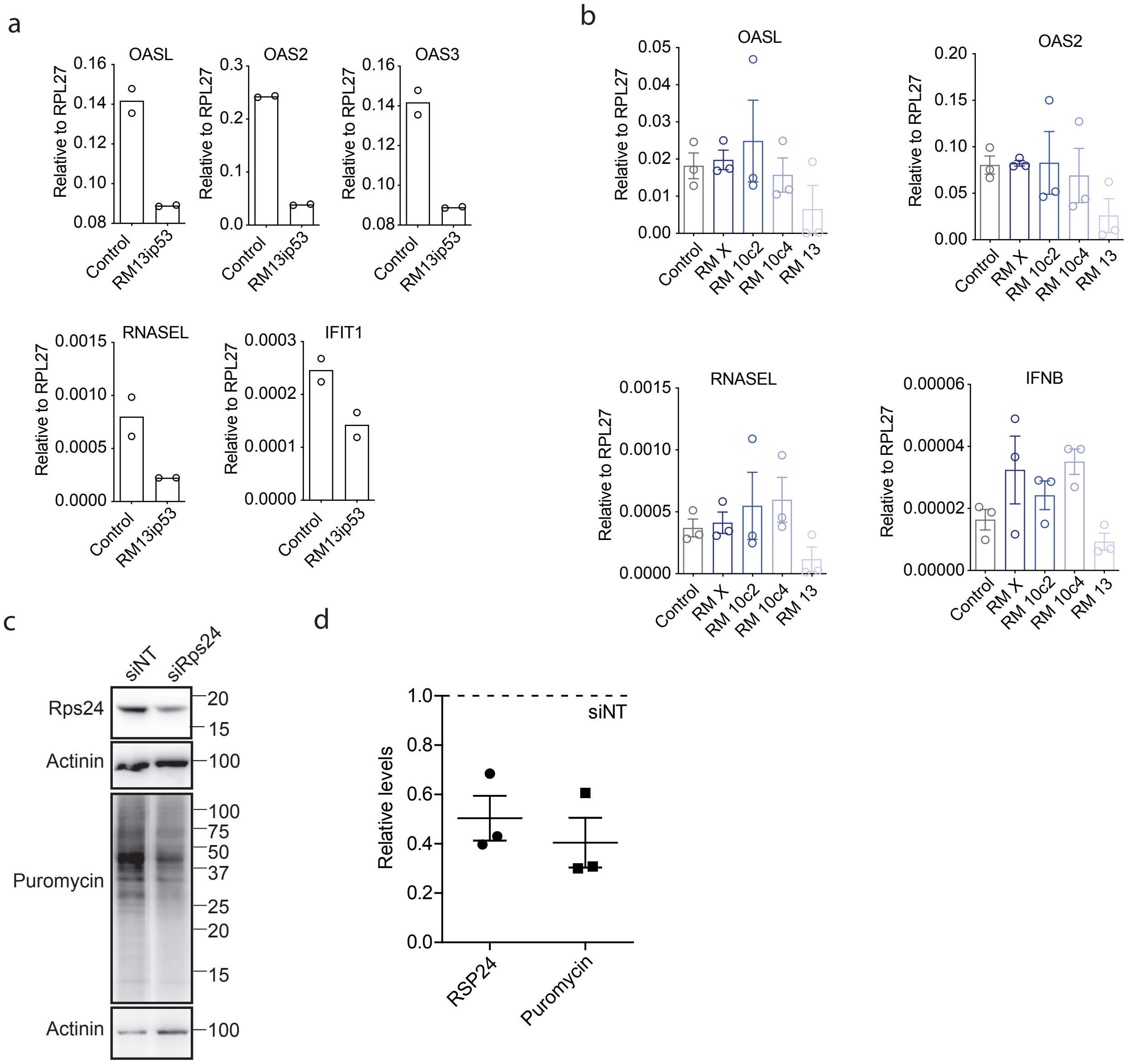

Supplementary figure 6 **Distinct and shared expression changes in response to monosomy**
a, b. Monosomic and control cells were treated with 100U of Interferon B in **(a)** and 1000U in **(b).** The expression of interferon reponse genes was quantified by qPCR. Bars dispaly the mean + SEM of 3 independent experiments in **b.**
c Representative blot of RPS24 levels upon siRNA for RPS24 and for non-targeting control (NT). Levels of RPS24 and puromycin incorporation are shown. Actinin was used as a loading control. **d**. Quantification of three independent experiments as in c. The expression of RPS24 and Puromycin in RPS24 transfected cells was normalized to NT control. Mean with SEM is shown. Source data are provided as a Source Data file.

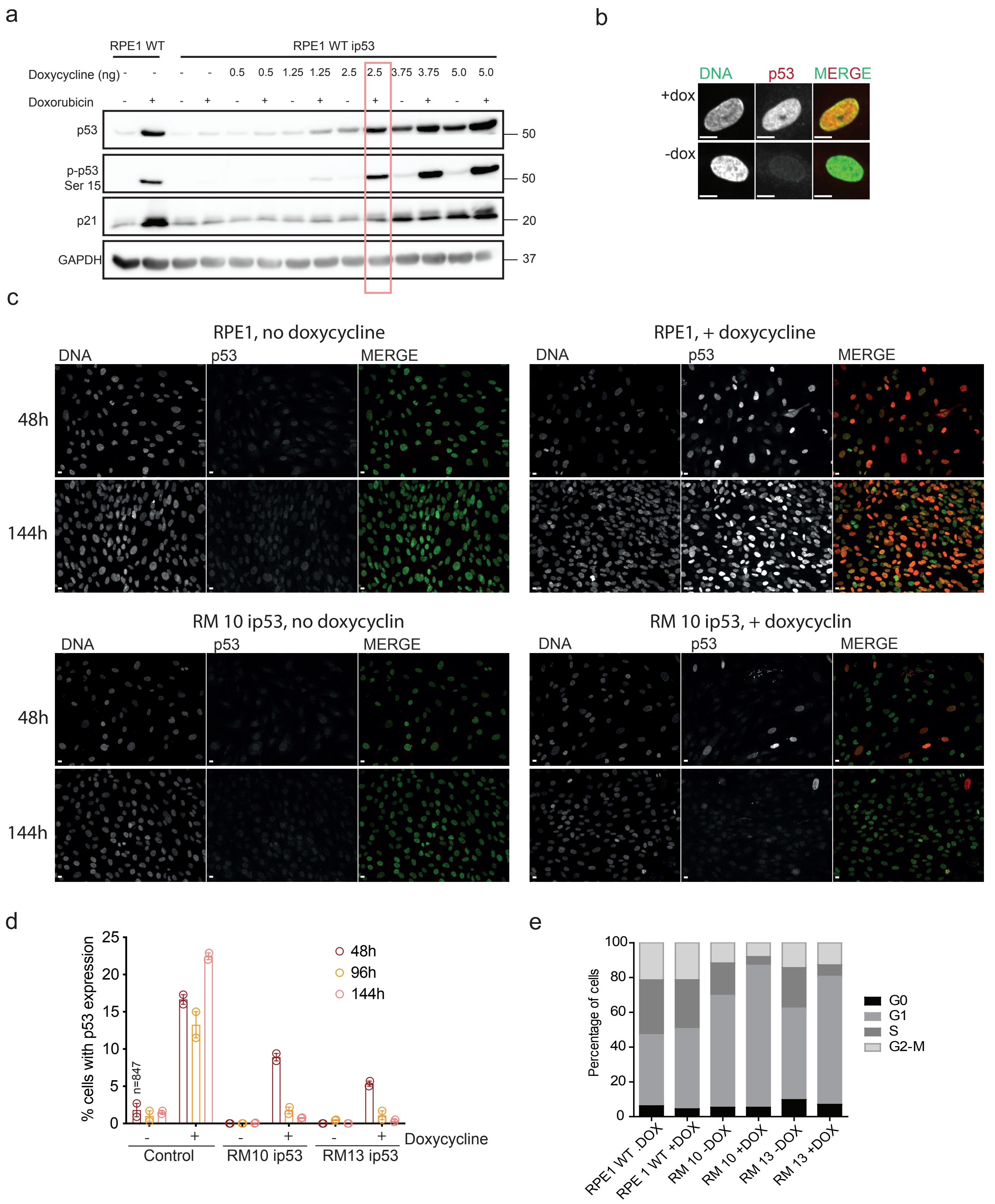

Supplementary figure 7 **Restoration of p53 in monosomic cell lines**
a. Titration of the doxycycline to reach normal p53 expression in RPE1 WT ip53 cell line. RPE1 WT is used as a control. Cells were treated with either doxycycline (in nanograms) or doxorubicin, or both. GAPDH serves as a loading control. Marked levels (red box) were used for the experiments. **b, c.** Immunofluorescence staining of p53 in cells with and without doxycycline treatment. DNA was stained with Sytox green, p53 is visualized in red. Scale bar - 10 µm. **d.** Quantification shows the percentage of p53 expressing cells with and without treatment with doxycycline. Bars display mean ± SEM of two independent experiments. Number of cells analyzed (Control no doxycyline 48h= 863, 96h=1677, 144h=2190; with doxycycline 48h= 656, 96h= 2515, 144h= 2307; RM10 ip53 no doxycyline 48h= 1042, 96h= 1179, 144h= 1632; with doxycycline 48h= 804, 96h= 791, 144h= 1559; RM13 ip53 no doxycyline 48h= 835, 96h= 1006, 144h= 1688; with doxycycline 48h= 1008, 96h= 1473, 144h= 2282) **e.** Cell cycle profile of monosomies with and without p53. Percentage of cells in different cell cycle phases were plotted. The representative gating strategy is shown in sup. fig 9. Source data are provided as a Source Data file.

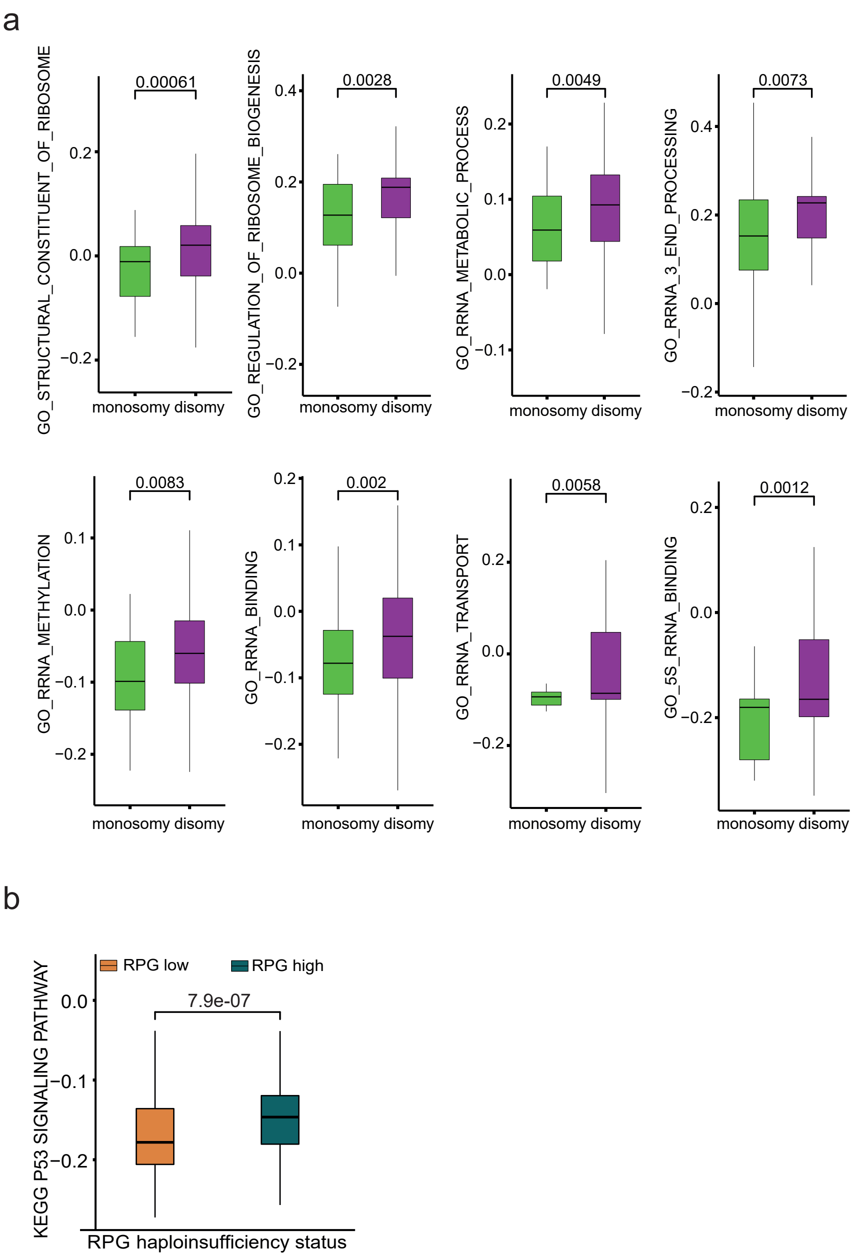

Supplementary figure 8 **CCLE transcriptome data comparing monosomy cell lines to disomy cell lines**

a. Transcriptomic analysis shows the ssGSEA enrichment score of GO terms related to ribosomes and rRNA in monosomic cell lines (n=48) compared to disomic cell lines (n=349). **b.** KEGG p53 pathway score of monosomy cell lines from CCLE. Monosomy cell lines were divided into two groups based on the median RPG abundance of the entire cohort. Orange box denotes group whose RPG expression is lower than the median of the cohort (n=188) and blue box represents the group with RPG expression higher than the median of the cohort (n=208). For both **a & b** One-sided Wilcoxon rank sum text was used to evaluate the statistical significance in all figures, p-values are shown in the respective plots. All box plots include the median line, the box denotes the interquartile range (IQR), whiskers denote the rest of the data distribution.

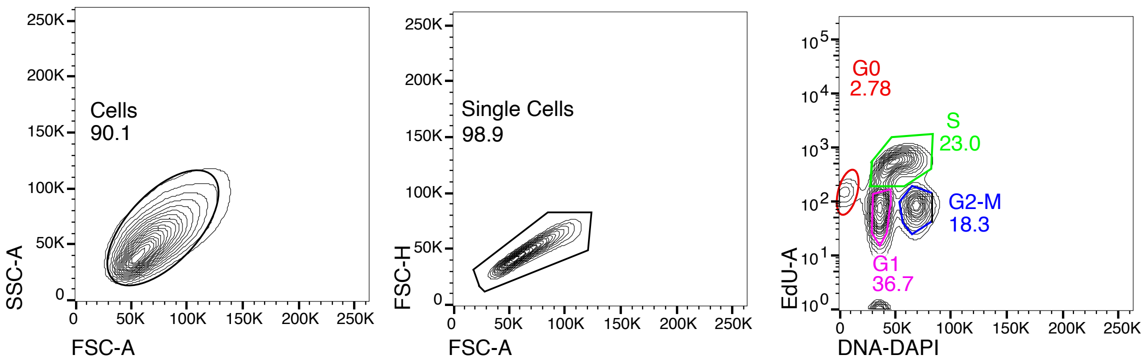

Supplementary figure 9. **FACS gating strategy for cell cycle phases.**
After obtaining the single cells, different phases of cell cycle are obtained by plotting DNA-DAPI and EdU signal. Each phase of the cell cycle (G0, G1, S and G2-M) are colored differently. Similar gating strategy was used in Supplementary figure 1b and Supplementary figure 7e.

Supplementary table 1 Engineered monosomic cell lines

Name	Parent	Altered chromosome	Fraction of monosomic cells	Remarks
RM10	RPE1-hTERT p53 -/-	10	Chr.10<br>100% (N=25)	This work
RM 10;18	RPE1-hTERT p53 -/-	10 and 18	Chr.10<br>100% (N=20)	This work
RM X	RPE1-hTERT p53 -/-	X	Chr. X<br>100% (N=15)	This work
RM 13	RPE1-hTERT p53 -/-	13	Chr. 13<br>91% (N=11)	This work
RM13 kd	RPE1-hTERT shRNA-p53; H2B-Dendra2	13	N/A	Soto et al, 2017
RM 7;10 kd	RPE1-hTERT shRNA-p53; H2B-Dendra2	7 and 10	N/A	Soto et al, 2017
RM 19p kd	RPE1-hTERT shRNA-p53; H2B-Dendra2	19p	N/A	Soto et al, 2017
RPE1 ip53	RPE1-hTERT p53 -/-		2N	This work
RM 13 ip53	RM 13	13	Chr.13<br>85% (N=13)	This work
RM 10 ip53	RM 10;18	10 and 18	Chr.10<br>75% (N=24)	This work

Fraction of monosomic cells:

Percentage of metaphase spreads having one copy of respective monosomic chromosome and 2 copies of diploid chromosome.

N= number of metaphase spreads

Supplementary Table 2 List of used antibodies and siRNA

**Antibodies**			
**Name of the protein**	**Company**	**Identification number**	**Dilution**
P53 (DO-1)	Santa Cruz	Sc-126	1:500
Anti puromycin 12D10	Merck Millipore	MABE343	1:1000
p21 Waf1/Kip1	Cell signaling	2947	1:1000
p-eIF2 alpha (Ser51)	Cell signaling	9721S	1:1000
eIF2 alpha	Cell signaling	9722S	1:1000
LC 3a/b	Cell signaling	4108	1:1000
p70 S6 Kinase	Cell signaling	2708	1:1000
p-p70 S6 Kinase	Cell signaling	9205	1:1000
Ribosomal Protein L21 (D7)	Santa Cruz	Sc-393663	1:200
Ribosomal Protein S24	Bethyl	A303-842A	1:2000
alpha-actinin	Santa Cruz	sc-17829	1:1000
HSP90	Cell signalling	4874	1:1000
HSP70/HSP72	Enzo	ADI-SPA-902	1:1000
Chk1	Abcam	Ab32531-100	1:1000
p-Chk1	Cell signalling	2348	1:1000
MCM2	Abcam	Ab4461	1:2000
MCM7	Santa Cruz	Sc9966	1:1000
pRPA32(s33)	Bethyl	A300-246A	1:1000
pRPA32(s4/s8)	Bethyl	IHC-00422	1:1000
RPA32	Abcam	Ab2175	1:1000
P62 lck ligand	BD Transduction	610832	1:1000
Cenp B	Santacruz	Sc376392	1:1000
γH2AX	Abcam	Ab2893	1:1000
Goat anti-rabbit HRP	R & D	HAF008	1:5000
Goat anti-mouse HRP	R & D	HAF007	1:5000
**siRNA**			
siRPL21	Dharmacon	M-012910-01-0005 (Smartpool)<br>Sequences:<br>GUACCUGGGUUCAACUAAA<br>GAGAAUUAAUGUGCGUAUU<br>GAGGAGAGGCACCCGAUAU<br>CCACAUAUAUGCGAAUCUA	
siGENOME Non-Targeting Control siRNA Pool #1	Dharmacon	D-001210-01-05(Smartpool)<br>Sequences:<br>UAGCGACUAAACACAUCAA<br>UAAGGCUAUGAAGAGAUAC<br>AUGUAUUGGCCUGUAUUAG<br>AUGAACGUGAAUUGCUCAA	

siGENOME siRPS24	Dharmacon	M-011155-01-0005 (Smartpool)<br>Sequences:<br>GAACGACACCGUAACUAUC<br>AGACAUGGCCUGUAUGAGA<br>CAACGAAAGGAACGCAAGA<br>GUACAAGACCACACCGGAU	

Supplementary Table 3 List of used primers

hOAS2 Fwd	AGGTGGCTCCTATGGACGGAA
hOAS2 Rev	GGCTTCTCTTCTGATCCTGGAATTG
hOASL Fwd	GCAGAAATTTCCAGGACCAC
hOASL Rev	CCCATCACGGTCACCATTG
hOAS3 Fwd	CCCTGGTCTGAGACTCACGTTT
hOAS3 Rev	GACTTGTGGCTTGGGTTTGAC
hRNASEL Fwd	GGCCTTCTGAACATTCCAAAAG
hRNASEL Rev	TCCGGATGAACTTTAGCAGATC
hIFIT1 Fwd	TACCTGGACAAGGTGGAGAA
hIFIT1 Rev	GTGAGGACATGTTGGCTAGA
hRPL27 Fwd	ATCGCCAAGAGATCAAAGATAA
hRPL27 Rev	TCTGAAGACATCCTTATTGACG
