# Supplementary material for: Systems approaches identify the consequences of monosomy in somatic human cells
Source: Nat Commun. 2021 Sep 22;12:5576. doi: 10.1038/s41467-021-25288-x (PMC8458293; doi:10.1038/s41467-021-25288-x)
Supplement: Supplementary file 3 — Description of Additional Supplementary Files [file 41467_2021_25288_MOESM3_ESM.pdf]

Description of Additional Supplementary Files

File Name: Supplementary Data 1
Description: Genome, transcriptome and proteome quantification

File Name: Supplementary Data 2
Description: Comparison of the gene dosage changes

File Name: Supplementary Data 3
Description: Differential pathway regulation in monosomic cells

File Name: Supplementary Data 4
Description: Gene expression changes in monosomic cells with and without functional p53

File Name: Supplementary Data 5
Description: Gene set enrichment analysis of tumors of different ploidy extracted from TCGA database

File Name: Supplementary Data 6
Description: Gene set enrichment analysis of cell lines of different ploidy extracted from CCLE database
