# Supplementary material for: Systems approaches identify the consequences of monosomy in somatic human cells
Source: Nat Commun. 2021 Sep 22;12:5576. doi: 10.1038/s41467-021-25288-x (PMC8458293; doi:10.1038/s41467-021-25288-x)
Supplement: Supplementary file 10 — Reporting Summary [file 41467_2021_25288_MOESM10_ESM.pdf]

nature portfolio

Corresponding author(s): Zuzana Storchova

Last updated by author(s): Jun 21, 2021

Reporting Summary

Nature Portfolio wishes to improve the reproducibility of the work that we publish. This form provides structure for consistency and transparency in reporting. For further information on Nature Portfolio policies, see our Editorial Policies and the Editorial Policy Checklist.

Statistics

For all statistical analyses, confirm that the following items are present in the figure legend, table legend, main text, or Methods section.

n/a	Confirmed	
☐	☒	The exact sample size (n) for each experimental group/condition, given as a discrete number and unit of measurement
☐	☒	A statement on whether measurements were taken from distinct samples or whether the same sample was measured repeatedly
☐	☒	The statistical test(s) used AND whether they are one- or two-sided<br>*Only common tests should be described solely by name; describe more complex techniques in the Methods section.*
☒	☐	A description of all covariates tested
☒	☐	A description of any assumptions or corrections, such as tests of normality and adjustment for multiple comparisons
☐	☒	A full description of the statistical parameters including central tendency (e.g. means) or other basic estimates (e.g. regression coefficient) AND variation (e.g. standard deviation) or associated estimates of uncertainty (e.g. confidence intervals)
☐	☒	For null hypothesis testing, the test statistic (e.g. F, t, r) with confidence intervals, effect sizes, degrees of freedom and P value noted<br>*Give P values as exact values whenever suitable.*
☒	☐	For Bayesian analysis, information on the choice of priors and Markov chain Monte Carlo settings
☒	☐	For hierarchical and complex designs, identification of the appropriate level for tests and full reporting of outcomes
☒	☐	Estimates of effect sizes (e.g. Cohen's d, Pearson's r), indicating how they were calculated

Our web collection on statistics for biologists contains articles on many of the points above.

Software and code

Policy information about availability of computer code

Data collection	Western blots were imaged using Azure c500<br>Promega Glomax Explorer microplate reader for measuring luminescence (Cell titer glo based assays)<br>Attune NxT Flow Cytometer (Thermo Fisher Scientific), Attune NxT Software 3.1.1243.0<br>Immunofluorescence images were captured using AxioObserver Z1 equipped with CSU-Xl spinning disk confocal head (Yokogawa) and Laser stack launch (31, Denver, CO), ABSOLUTE algorithm.
Data analysis	Graph prism 5 and 9,Fiji lmage J 1.51F,MaxQuant vl.6.3.3,Perseus vl.6.8, R v3.6.1,R package: ggplot2 v3.2.0,R package: dplyr v0.8.3<br>R package: reshape2 vl.4.3,R package: gtools v 3.8.1,R package: EQL vl.0-1,R package: kdensity vl.0.1, STAR alinger v2.5.2a,DEseq2 1.14.1,Bioconductor 3.6.1, Illumina BaseCaller, BCL2Fastq v2.20.0.422<br>R package:<br>Limma v3.42.2,R package:VSN v 3.54.0,ClustVis, Slidebook 6

For manuscripts utilizing custom algorithms or software that are central to the research but not yet described in published literature, software must be made available to editors and reviewers. We strongly encourage code deposition in a community repository (e.g. GitHub). See the Nature Portfolio guidelines for submitting code & software for further information.

Data

Policy information about availability of data

All manuscripts must include a data availability statement. This statement should provide the following information, where applicable:

- Accession codes, unique identifiers, or web links for publicly available datasets
- A description of any restrictions on data availability
- For clinical datasets or third party data, please ensure that the statement adheres to our policy

Source Data is provided with this paper.
The TMT label mass spectrometry proteomics data have been deposited to the ProteomeXchange Consortium via the PRIDE partner repository with the dataset identifier PXD018440[http://www.ebi.ac.uk/pride/archive/projects/PXD018440]
LFQ data was deposited on PRIDE partner repository with the dataset identifier
PXD022927 [http://www.ebi.ac.uk/pride/archive/projects/PXD022927]
The transcriptome of RM X was uploaded on ENA with accession number
PRJEB38328 [https://www.ebi.ac.uk/ena/browser/view/PRJNA633154]
All other transcriptomes are uploaded in NCBI Omnibus with accession number
GSE150686 [https://www.ncbi.nlm.nih.gov/geo/query/acc.cgi?acc=GSE150686]
The human ribosome cryo-EM data used fort he ribosome structure presentation
6Y2L [http://doi.org/10.2210/pdb6Y2L/pdb]
The TCGA data used for the analysis are depositited at
[https://api.gdc.cancer.gov/data/5dd5a767-8f9f-4579-abee-b1306a4d0ad2]
The CCLE data use for the analysis are deposited at
[https://data.broadinstitute.org/ccle/CCLE_ABSOLUTE_combined_20181227.xlsx]
The Aneuploidy Scores used for the analysis are deposited at
[https://www.sciencedirect.com/science/article/pii/S1535610818301119#app2]

Field-specific reporting

Please select the one below that is the best fit for your research. If you are not sure, read the appropriate sections before making your selection.

☒ Life sciences ☐ Behavioural & social sciences ☐ Ecological, evolutionary & environmental sciences

For a reference copy of the document with all sections, see nature.com/documents/nr-reporting-summary-flat.pdf

Life sciences study design

All studies must disclose on these points even when the disclosure is negative.

Sample size	No study size calculation was performed and the sample size was based on our prior studies using the same types of assays and published literature to ensure statistically significant results. All key experiments were repeated independently using different cell lines or different techniques. For in vitro studies n=3 was used as a standard sample size.
Data exclusions	No data was excluded from the analysis
Replication	All the experiments were performed from at least 3 independent experiments (unless stated otherwise), the reproducibility was statistically evaluated. All attempts at replication were successful.
Randomization	No randomization was applied as cells with monosomic karyotype were classified as Monosomies and diploid as control.
Blinding	The experimenters were blinded using well number for the analysis of live cell imaging. For the anaphase bridges and micronuclei quantification and DNA damage analysis the experimenters were not blinded. The application of treatments and processing procedures negated the possibility of blinding but there was no human bias given all data was collected independently using instrumentation.

Reporting for specific materials, systems and methods

We require information from authors about some types of materials, experimental systems and methods used in many studies. Here, indicate whether each material, system or method listed is relevant to your study. If you are not sure if a list item applies to your research, read the appropriate section before selecting a response.

Materials & experimental systems

n/a	Involved in the study
☐	☒ Antibodies
☐	☒ Eukaryotic cell lines
☒	☐ Palaeontology and archaeology
☒	☐ Animals and other organisms
☒	☐ Human research participants
☒	☐ Clinical data
☒	☐ Dual use research of concern

Methods

n/a	Involved in the study
☒	☐ ChIP-seq
☐	☒ Flow cytometry
☒	☐ MRI-based neuroimaging

Antibodies

Antibodies used	P53 (DO-1) (Santa cruz-126),Anti puromycin 12D10 (Millipore-MABE343),p21 Waf1/Kip1 (Cell signalling 2947),p-eIF2 alpha (Cell signalling Ser51) (9721S),eIF2 alpha (Cell signalling 9722S),LC 3 I/II (Cell signalling 4108),p70 S6 Kinase (Cell signalling 2708),p-p70 S6 Kinase (Cell signalling 9205),Ribosomal Protein L21 (D7) (Santa cruz-393663), Ribosomal Protein S24 (Bethyl A303-842A)alpha-actinin (Santa cruz-17829),HSP90 (Cell signalling 4874), HSP70/HSP72 (Enzo ADI-SPA-902),Chk1 (Abcam 32531-100),p-Chk1 (Cell signalling 2348), MCM2 (Abcam 4461), MCM7 (Santa cruz 9966), pRPA32(s33) (Bethyl A300-246A), pRPA32(s4/s8) (Bethyl- IHC-00422),RPA32 (Abcam 2175),P62 lck ligand (BD biosciences 610832), Cenp B (Santa cruz 376392), gammaH2AX (Abcam 2893), Goat anti-rabbit HRP (R & D HAF008) , Goat anti-mouse HRP (R & D HAF007). The dilutions are listed in sup. table 6
Validation	For western blot and immunofluorescence, all commerically available antibodies were used according to the validations performed by the manufacturers. For the DNA damage analysis by western blotting, cells treated with hydroxyurea was used as positive control. For the anti-puromycin antibody, cells treated with cyclohexamide was used to validate the antibody. LC3 I/II and p62 antibodies were validated by lysates treated with Bafilomycin.p53 and p21 antibodies were validated by p53 knockout cell lysates and cells treated with Doxorubicin and Nutlin 3a.

Eukaryotic cell lines

Policy information about cell lines

Cell line source(s)	hTERT RPE-1 cells (46,XX), HEK293T were purchased from ATCC® (CRL-4000). RPE TP53 Knockout cells were generated in this study with sgRNAs or TALENS against TP53. Monosomic cell lines were derived from RPE1 p53 KO. The detailed description of generation of monosomic cell lines are described in details in methods section. Supplementary table 1 list all the cell lines used in this study.
Authentication	No authentication as such was performed. However, gain of chromosome 10q, a known karyotype characteristic of RPE1 cells was also observed in our cell lines. This finding was validated using whole genome sequencing and whole chromosomal painting .
Mycoplasma contamination	All the cell lines are tested negative for mycoplasma contamination using PlasmoTest (InvivoGen)
Commonly misidentified lines (See ICLAC register)	No commonly misidentified cell lines were used in this study

Flow Cytometry

Plots

Confirm that:

☒ The axis labels state the marker and fluorochrome used (e.g. CD4-FITC).

☒ The axis scales are clearly visible. Include numbers along axes only for bottom left plot of group (a 'group' is an analysis of identical markers).

☒ All plots are contour plots with outliers or pseudocolor plots.

☒ A numerical value for number of cells or percentage (with statistics) is provided.

Methodology

Sample preparation	Proliferating control and the monosomic cells were labelled with EdU 30 min before harvesting. Cells were fixed and permeabilized for 15 min with Fix perm (Thermo Fisher scientific), followed by incubation with EdU Click-iT cocktail (Invitrogen) as per the manufacturer's instructions. Cells were resuspended in PBS containing RNase (10 µg/mL) and DAPI.
Instrument	Attune NxT acoustic focusing flow cytometer
Software	Attune NxT Software 3.1.1243.0

Cell population abundance	Total cells:>80%, Single cells: > 90%
Gating strategy	1st gate FSC-A/SSC-A, 2nd gate FSC-A/FSC-H, 3rd gate DAPI+EdU. DAPI for DNA content and EdU for labelling S phase cells. 2N DNA content witout EdU (Gl phase), 4N DNA content without EdU (G2/M phase) and EdU positive cells of 2N and 4N DNA content (S phase). Supplementary figure 9.

☒ Tick this box to confirm that a figure exemplifying the gating strategy is provided in the Supplementary Information.
